# Characterisation of Flavonoid Aglycones by Negative Ion Chip-Based Nanospray Tandem Mass Spectrometry

**DOI:** 10.1155/2012/259217

**Published:** 2012-02-21

**Authors:** Paul J. Gates, Norberto P. Lopes

**Affiliations:** ^1^School of Chemistry, University of Bristol, Cantock's Close, Bristol BS8 1TS, UK; ^2^Faculdade de Ciências Farmacêuticas de Ribeirão Preto, Universidade de São Paulo, Via do Café S/N, 14040-903 Ribeirão Preto, SP, Brazil

## Abstract

Flavonoids are one of the most important classes of natural products having a wide variety of biological activities. There is wide interest in a range of medical and dietary applications, and having a rapid, reliable method for structural elucidation is essential. In this study a range of flavonoid standards are investigated by chip-based negative ion nanospray mass spectrometry. It was found that the different classes of flavonoid studied have a combination of distinct neutral losses from the precursor ion [M-H]^−^ along with characteristic low-mass ions. By looking only for this distinct pattern of product ions, it is possible to determine the class of flavonoid directly. This methodology is tested here by the analysis of a green tea extract, where the expected flavonoids were readily identified, along with quercetin, which is shown to be present at only about 2% of the most intense ion in the spectrum.

## 1. Introduction

Flavonoids are an important class of dietary natural products with a range of biological activities, such as antioxidant, UV-protection, antiparasitic, anti-inflammatory, and antifungal [1–6]. The flavonoids are subcategorised into eight different classes with some of the compounds also exhibiting possible beneficial properties such as health-promoting and anticancer activities [7]. The common C6-C3-C6 structural core for all flavonoids arises from the shikimate (C6-C3) and acetate (C6) biosynthetic pathways. In their review, Williams and Grayer pointed out that the theoretical number of possible flavonoid structures (with hydroxyl, methoxyl, methyl, isoprenyl benzyl, and sugar substituents) is enormous, and many new natural flavonoids are still to be isolated [8]. Until now, more than 9000 different flavonoids have been isolated. The majority were isolated and identified employing classical phytochemical procedures, and there is no doubt that many more new flavonoids remain to be discovered [8].

Many analytical methodologies have been developed to detect and quantify flavonoids, mostly using high-performance liquid chromatography with UV-VIS spectral detection. However, identification of flavonoids, as well as other natural products, through hyphenated systems (LC-UV) is limited since a complete chromatographic resolution for all chromophores is required to be sure that the correct conclusion is reached [9, 10]. Mass spectrometry (MS) with electrospray ionisation (ESI) has emerged as a complementary method for high sensitivity, selectivity, and fast analysis of natural products [11], such as sesquiterpene lactones [12] and alkaloids [13]. Among all mass spectrometry techniques, electrospray ionisation tandem mass spectrometry (ESI-MS/MS) using low-energy collision-induced dissociation (CID) has been the technique of choice for such studies through the technique's ability to analyse natural products with medium to high polarities [14].

Nanospray ionisation is an improvement over traditional ESI for the analysis of low volume low concentration samples [15]. With nanospray, it is possible to obtain mass spectra from picogram quantities of material with little sample clean-up being required. Standard nanospray uses disposable tips and as a result has problems with signal reproducibility between tips and difficulties with coupling to HPLC. With the development of automated “chip-based” nanospray systems, using arrays of uniform nanospray needles, the technique is becoming much more important [16]. In “chip-based” nanospray, the analyte solution is sprayed from a conductive pipette tip pressed against the rear of the chip using a small gas pressure and low voltage to create the spray. Each nanospray needle in the array is used only once to avoid contamination.

**Figure 1:**
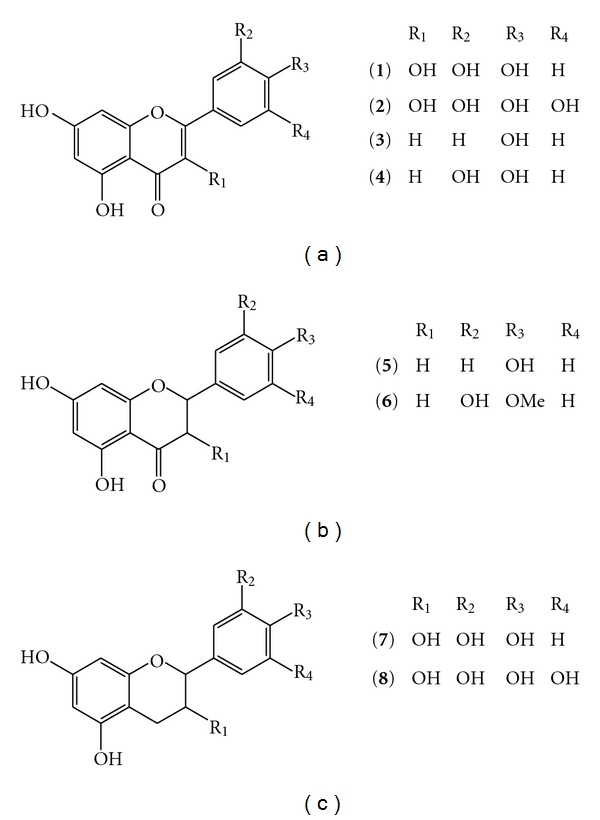
The structures of the flavonoids analysed. (**1**) Quercetin (molecular weight = 302); (**2**) Myricetin (molecular weight = 318); (**3**) Apigenin (molecular weight = 270); (**4**) Luteolin (molecular weight = 286); (**5**) Naringenin (molecular weight = 272); (**6**) Hesperetin (molecular weight = 302); (**7**) Catechin (molecular weight = 290) and (**8**) Epigallocatechin (molecular weight = 306).

In recent years, nanospray ionisation has been applied to the analysis of natural products, but there are still some doubts about the applicability of the technique for the analysis of small molecules. Analysis of retinal, carotenoids, and xanthophylls showed some significant differences between the ions observed between nanospray and electrospray ionisation [14, 17, 18]. These results could be correlated to differences in the source design and ionisation conditions for nanospray and open up a new area of research in natural product chemistry. Based upon these previous studies and the increasingly recognised importance of flavonoids in the human diet along with the increase in metabolomic studies, the purpose of this study is to establish a sound basis for the ionisation and fragmentation of four aglycone flavonoid classes (Figure 1) in negative ion nanospray ionisation. The application and power of the technique to “real world” samples is exemplified with the identification of medium-polarity flavonoids from a simple extract of green tea without employing any prior sample preparation, clean-up, or chromatography.

## 2. Experimental

### 2.1. Materials

The flavonoid standards (Figure 1) were isolated as previously described [19] or obtained from Sigma-Aldrich (United Kingdom). Solutions of the analytes (approximetely 0.1 mg/mL) in 100% HPLC-grade methanol (Fisher Scientific) were prepared immediately prior to the analysis. The green tea sample was obtained from a local supermarket. A few grains were dissolved in 100% methanol with the sample centrifuged (13,000 rpm, 5 mins) prior to the analysis.

### 2.2. Instrumentation

Nanospray ionisation analyses were performed on a QStar-XL quadrupole-time-of-flight hybrid instrument (Applied Biosystems, Warrington, UK) using a NanoMate HD automatic chip-based nanospray system (Advion Biosciences, Norwich, UK). Instrument control, data acquisition, and data processing were performed through the Analyst QS version 1.1 software (Applied Biosystems, Warrington, UK). NanoMate control was through the ChipSoft software (Advion Biosciences, Norwich, UK). The NanoMate was set for 5 *μ*L of solution to be aspirated and sprayed through a NanoMate 400 chip at 1.45 kV with a nitrogen back pressure of 0.4 psi. QStar acquisition parameters were ion source gas flow rate, 50; curtain gas flow rate, 20; ion spray voltage, 2700 V; declustering potential, 75 V; focusing potential, 280 V; declustering potential 2, 15 V. CID-MS/MS was performed at a collision energy in the range from −20 to −40 eV. The ion source gas, curtain gas, and collision gas were all nitrogen.

## 3. Results and Discussion

**Figure 2:**
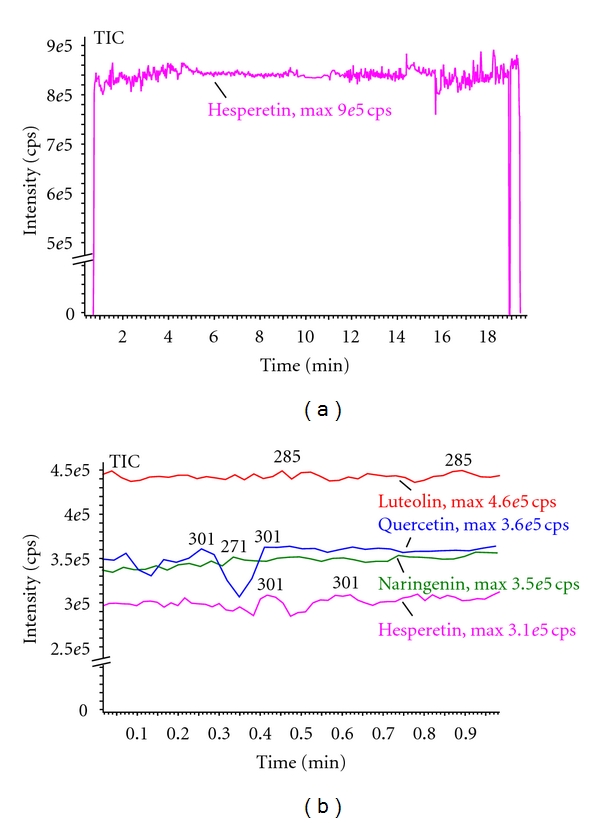
Demonstration of the stability of the chip-based nanospray infusion. The data shows plots of total ion count (from 5 *μ*L aspirations) versus time for [M-H]^−^ ions. Plot (a) is for hesperetin over a 20 minute run. The onset of nanospray is at about 30 s into the run with about 15 minutes of highly stable spray. After 15 minutes, the spray is less stable until the spray breaks down at about 19.5 minutes. Plot (b) is of luteolin, quercetin, naringenin, and hesperetin over a 1-minute run demonstrating intersample reproducibility.

The compounds quercetin (flavonol, **1**), apigenin (flavone, **3**), naringenin (flavanone, **5**), and hesperetin (flavanone, **6**) (Figure 1) were used as standards to study their ability to produce high-intensity, stable-deprotonated molecule signals in negative ion mode nanospray ionisation. 100% HPLC methanol proved to be an excellent solvent for these studies with stable ion signals being produced for up to 20 minutes (Figure 2). This is essential as it allows for a number of tandem mass spectrometry (MS/MS) experiments to be performed on the same sample without any adjustments or tuning of the nanospray source. Use of methanol resulted in no observed methylation reactions as has previously been described for other natural products [20]. Over the range of source conditions used, all the aglycone flavonoids produced an intense and stable spray for at least 15 minutes from single 5 *μ*L analyte solution aspirations. This demonstrates the possibility to work with more complex flavonoid samples and allows for setting up automatic MS/MS acquisitions from a batch analysis.

**Figure 3:**
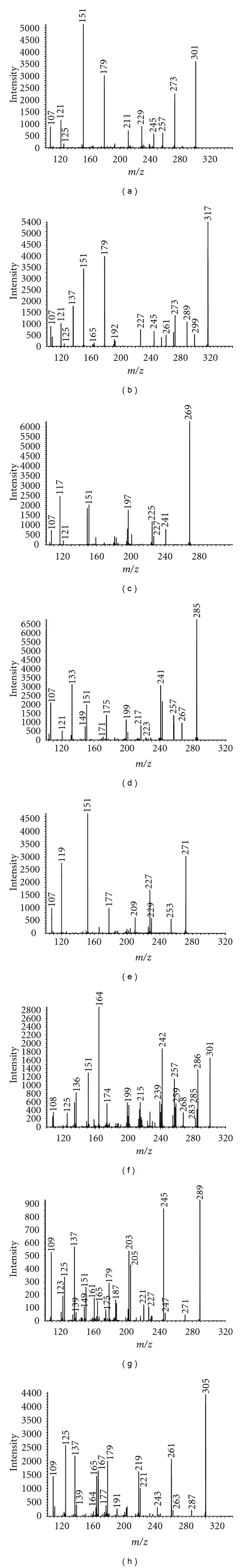
The negative ion nanospray product ion spectra of the eight flavonoids studied. Spectrum (a) is of quercetin, 1: (precursor ion (PI) *m/z* 301), (b) myricetin, 2: (PI *m/z* 317), (c) apigenin, 3: (PI *m/z* 269), (d) luteolin, 4: (PI *m/z* 285), (e) naringenin, 5: (PI *m/z* 271), (f) hesperetin, 6: (PI *m/z* 301), (g) catechin, 7: (PI *m/z* 289), and (h) epigallocatechin 8: (PI *m/z* 305).

Following on from the ion formation studies, the systematic investigation was continued to determine the best CID collision energies required for effective product ion formation whilst eliminating unwanted gas-phase interactions. Collision energies from −20 to −40 eV resulted in good product ion spectra, with, as expected, more product ions being observed at higher voltages (more negative). A collision energy of around −35 eV was determined to result in the “best” product ion spectra (Figure 3). Examination of the spectra revealed high levels of complexity with many competing fragmentation routes. The main neutral molecules lost from the [M-H]^−^ ions consisted of a combination of H_2_O, CO, CO_2_, and/or H_2_CCO (Figure 3). A detailed analysis of all the spectra indicates that a combination of a specific order of neutral eliminations occurs along with the presence of a series of diagnostic low-mass product ions for each of the flavonoid classes analysed (Table 1 and Figure 3) resulting in the quick and reliable method for the identification of the flavonoid class. The diagnostic low-mass product ions result from ring contraction reactions which follow the same mechanisms as previously reported for flavonoids in negative mode ESI [21]. All of the flavonoids (except the flavanols) have the previously described ions at *m/z* 151 and 107 [21], whereas the flavanols catechin and epigallocatechin (with no oxidation at carbon 3, but following a similar ring contraction mechanism) result in the product ions at *m/z* 137 and 109. Also, all of the flavonoids except the flavones have an ion at *m/z* 125, and the flavones have an ion at *m/z* 121.

**Figure 4:**
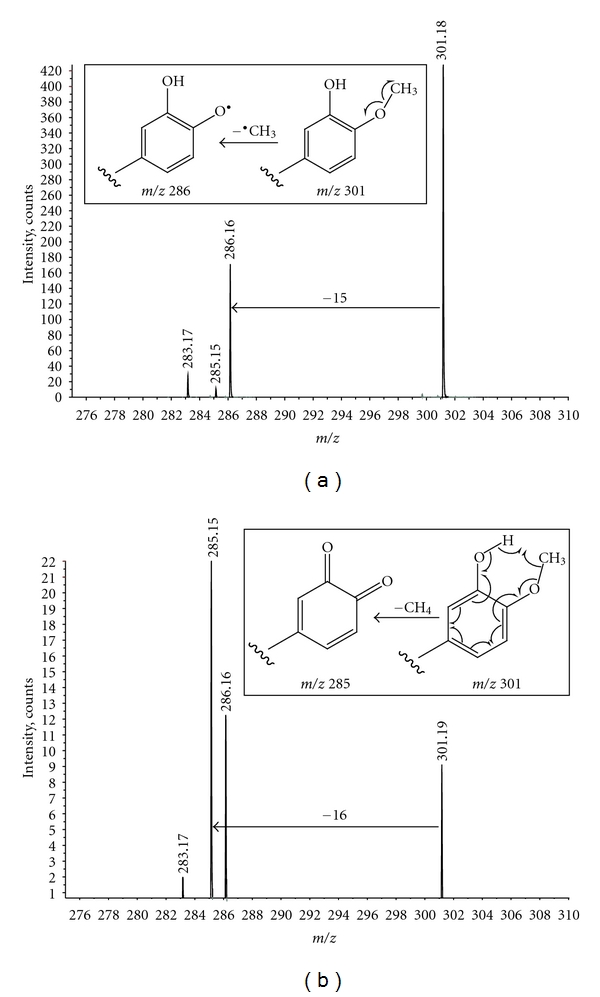
Enlargements of negative ion nanospray product ion spectra of hesperetin at low (a) and high (b) collision energies. The competition between losses of ^•^CH_3_ and CH_4_ is clearly observed. At higher collision energy, the radical ion (*m/z* 286) has fragmented further to leave the quinonic ion (*m/z* 285) intact. The mechanism of formation of the two ions is shown in the inserts.

The flavanone hesperetin has a methoxyl substitution at the aromatic ring and showed elimination of a methyl radical (^•^CH_3_) similar to that previously reported for mycosporine-like amino acids [22] and some other flavonoids [23]. Observation of this behaviour in nanospray allows the easy distinguishing of methoxylated flavonoids with identical molecular mass, for example, when screening plant extracts for flavonoid composition as previously report in ESI [23]. Increasing the collision energy for hesperetin results in an almost complete fragmentation of the radical ion, but allows for the observation of a loss of 16 mass units. An unusual CH_4_ elimination has been previously described for heterocyclic aromatic amines which is proposed to be due to a gas-phase ion-molecule aromatic-nucleophilic substitution between *β*-carbolines and water vapour [24]. With hesperetin, the loss of 16 is suggested to be due to CH_4_ elimination involving the methoxyl group and the *ortho*-hydroxyl group.  Figure 4 shows the expansion of two product ion spectra of hesperetin at different collisional energies, clearly showing the competing losses of ^•^CH_3_ and CH_4_. The mechanism for loss of ^•^CH_3_ proceeds through homolytic cleavage as previously described [22, 23]. The mechanism for water elimination from ortho-substituted aromatic esters is well known in electron ionisation. In this case we suggest that a similar cyclic rearrangement through homolytic cleavage is occurring, but involving the hydroxyl substitution, resulting in a stable quinonic ion (Figure 4). Both of these mechanisms, when taken together, are very useful for the structure elucidation of disubstituted flavonoids.

**Figure 5:**
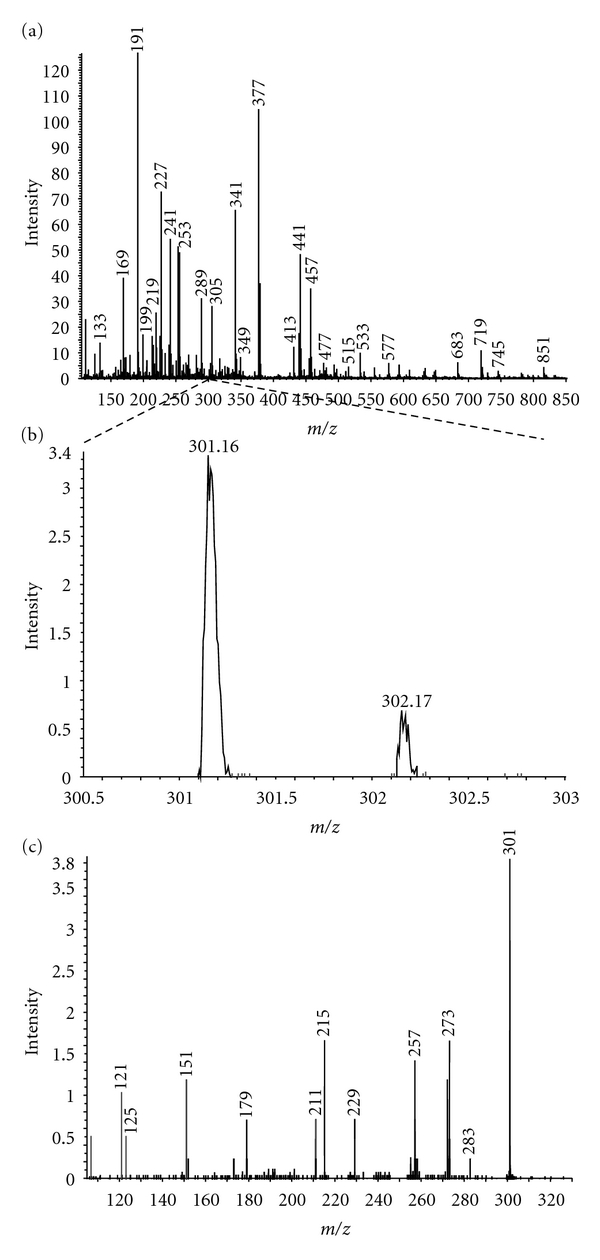
Negative ion nanospray spectra of the green tea extract. Spectrum (a) is the total extract recorded over a wide *m/z* range. Spectrum (b) is an enlargement of (a) to show the peak at *m/z* 301 at approximately 2% of the intensity of the most intense ion in spectrum (a). Spectrum (c) is the product ion spectrum of *m/z* 301 which clearly demonstrates the sensitivity of the technique.

The analysis of a green tea extract in methanol was performed to demonstrate the utility of the technique. The analysis was performed without any chromatography or sample cleanup. The negative ion nanospray spectrum (Figure 5) is very complicated with a considerable number of ions over a wide mass range. Some of the observed masses (*m/z* 289, 305 and 317) match to the flavonoid standards already analysed in this study, and analysis of the MS/MS spectra (data not shown) of these proved them to be the expected flavonoids present in green tea: catechin, **7**, (flavanol), epigallocatechin, **8**, (flavanol) and myricetin, **2**, (flavonol). Other intense peaks (*m/z* 441 and 457) are gallate flavonoids not considered in this initial study. To test the detection limit of the technique, the peak at *m/z* 301 was studied further (see Figure 5). This peak occurs at approximately 2% of the most abundant ion in the spectrum, but performing MS/MS for about 1 minute still produced a good intensity product ion spectrum (Figure 5). A thorough study of this spectrum reveals an almost identical series of peaks to that of the flavonol quercetin, **1** (Figure 2). The differences between the two spectra are probably down to the different collision energies used. Quercetin is one of the most biologically active flavonoids and is more normally found in citrus fruits. The confirmation of the presence of quercetin in green tea (even at the low levels in this particular sample) is a highly significant result and a powerful demonstration of the sensitivity and application of this methodology.

## 4. Conclusions

In this initial study, the application of chip-based negative ion nanospray is demonstrated for the analysis of a series of flavonoid standards. The best spectra where produced from 100% HPLC methanol. MS/MS analysis of four of the classes of flavonoids have shown that they have a different, characteristic sequences of neutral losses from their corresponding [M-H]^−^ precursor ions in combination with distinctive lower mass product ions. The application of this methodology is demonstrated for the analysis of a green tea extract where the expected flavonoids (catechin, epigallocatechin, and myricetin) were easily identified, along with the unexpected presence of quercetin (at approximately 2% of the most intense ion).

## Figures and Tables

**Table 1:** Table of the characteristic sequences of neutral losses, from their corresponding [M-H]^−^ precursor ions and characteristic low-mass product ions, for the four flavonoid classes analysed in this study.

Flavonoid class	Characteristic neutral losses	Characteristic productions
Flavonols (**1** and **2**)	−28, −44, −18	151, 125, 107
Flavones (**3** and **4**)	−28, −44, −44, −28, −42	151, 121, 107
Flavanones (**5** and **6**)	−18, −44, −44, −18, −42	151, 125, 107
Flavanols (**7** and **8**)	−18, −44, −44, −18, −42	137, 125, 109
